# Prevalence and risk factors of depression, anxiety, and stress among the Bangladeshi construction workers: A cross-sectional study

**DOI:** 10.1371/journal.pone.0307895

**Published:** 2024-08-19

**Authors:** Nitai Roy, Kallol Deb Paul, Sumaiya Sultana Tamanna, Anup Kumar Paul, Moneerah Mohammad Almerab, Mohammed A. Mamun

**Affiliations:** 1 Department of Biochemistry and Food Analysis, Patuakhali Science and Technology University, Patuakhali, Bangladesh; 2 Faculty of Nutrition and Food Science, Patuakhali Science and Technology University, Patuakhali, Bangladesh; 3 Department of Psychology, College of Education and Human Development, Princess Nourah Bint Abdulrahman University, Riyadh, Saudi Arabia; 4 CHINTA Research Bangladesh, Dhaka, Bangladesh; 5 Department of Public Health and Informatics, Jahangirnagar University, Dhaka, Bangladesh; 6 Department of Public Health, University of South Asia, Dhaka, Bangladesh; Jashore University of Science and Technology (JUST), BANGLADESH

## Abstract

**Background:**

Construction workers are a population that is at risk for mental illnesses such as depression, anxiety, and even suicide due to the high stress and physical demands of their work. This study aimed to determine the prevalence and risk factors for depression, anxiety, and stress among Bangladeshi construction workers.

**Methods:**

From February 2022 to June 2022, community-based cross-sectional research was conducted among construction workers. Survey data was gathered using interviewer administered questionnaires with 502 participants from the construction sites. Data were collected based on the information related to socio-demographics, lifestyle, occupation, health hazards, and mental health (i.e., depression, anxiety, and stress). The results were interpreted using the chi-square test and logistic regression utilizing SPSS statistical software.

**Results:**

The study revealed the prevalence rates of depression, anxiety, and stress among construction workers to be 17.9%, 30.3%, and 12%, respectively. Key findings indicate that construction workers who maintained a healthy sleep duration were 64% less likely to be depressed compared to those with poor sleep (AOR = 0.36; 95% CI: 0.21–0.61, *p*<0.001). Workers who did not experience breathing issues upon starting construction work had a 45% lower likelihood of experiencing depression (AOR = 0.55; 95% CI: 0.32–0.97, *p* = 0.037) and an 82% lower likelihood of experiencing anxiety (AOR = 0.18; 95% CI: 0.11–0.30, *p*<0.001). Bricklayer construction workers were 72% less likely to experience stress (AOR = 0.28; 95% CI: 0.08–0.95, *p* = 0.041), and workers without breathing issues after starting construction work were 66% less likely to experience stress (AOR = 0.34; 95% CI: 0.17–0.66, *p* = 0.001).

**Conclusions:**

The study found that depression, anxiety, and stress are prevalent among construction workers in Bangladesh, with breathing issues as a significant risk factor. Thus, there is a need for effective measures to reduce these problems and provide a safe working environment for construction workers to ensure their productivity and the country’s overall growth.

## Introduction

The construction industry is vital to any country’s economic growth and development. The sector has been expanding in Bangladesh since 1990, with large investments in multipurpose building projects, industrial developments, residential constructions, and infrastructure expansions. The sector is becoming increasingly important in the country’s economy, with 3.43 million construction employees and 4000 construction enterprises, contributing 7.5% to GDP in 2017–18 [1]. However, despite the importance of construction workers to the economy, it has become one of the leading sectors that cause mortality and disability worldwide. In Bangladesh, it accounts for 16% of all occupational fatalities, that is, about 100 deaths, along with an additional 100 injuries every year [2]. Though there are regulations in place to protect construction workers, such as the Bangladesh National Building Code 2006 and the Bangladesh Labor Act 2006, building site accidents still frequently occur due to a lack of oversight and law enforcement [2, 3]. Construction site safety and health remain a low priority, leading to serious injuries or fatalities among workers.

A construction manual worker, also known as a general or blue-collar worker in the construction sector, primarily operates on construction sites [4, 5]. They are typically involved in the practical aspects of the industry, focusing on tasks that do not involve design or finance. This category includes individuals such as manual laborers, armature fixers, interior finishers, plumbers, as well as skilled craftsmen like builders, electricians, carpenters, and bricklayers [6]. The construction industry can be a challenging and dangerous environment for workers, with long hours, monotony, and the potential for unemployment due to transitory and cyclical projects [7]. These factors can negatively impact the physical and emotional well-being of workers. Studies have shown that occupational stress can lead to physical and mental health issues. It is important to monitor psychological risk factors to understand their effects on worker health and well-being to reduce workplace injuries, prevent disability, and increase productivity [8]. Poor psychological health is prevalent in the construction sector worldwide, with an increased risk of depression, anxiety, suicidality, and suicide among workers [8–12]. Depression and anxiety are the most studied mental health illnesses in the construction sector, focusing on manual labor. Studies have found high rates of depression and anxiety among manual laborers in various countries, including Korea [13], the Netherlands [8], and Vietnam [14]. In addition, the mental health problems of other types of construction professionals such as site managers were also investigated and high levels of anxiety and depression were found in the United Kingdom [15].

Although the larger construction mental health literature is still developing, a wide range of factors has been investigated, including existing chronic conditions [16], young age [10], poor family support [17], long working hours [18], and other industry-related hazards [19]. Also, poor mental health has been linked to early physical, mental, emotional, social, and professional development stages [20]. Unhealthy industry culture (such as bullying, using drugs as a coping mechanism, etc.) [21, 22], unfavorable working conditions, and stressful construction work [10] can also contribute to poor mental health. Workers in both developed and developing countries experience various types of work-related stress. Studies have shown that young workers, such as construction professionals, are at a higher risk for psychological discomfort and social strain than older workers [10, 23]. Exposure to serious construction accidents can also lead to severe post-traumatic stress disorder [19], it can even lead to suicide [24]. It is important to address the aforementioned mental health illnesses affecting construction workers, but in developing countries like Bangladesh, little is known about the specific challenges these workers face.

Despite the growing discussion on the topic, research on the mental health of construction workers in developing countries is limited, which can aid in selecting effective therapies or measures for employees. A comprehensive assessment of the construction industry’s psychosocial work environment and common mental health problems is needed to understand the extent of psychosocial risk factors and mental health impacts in these jobs. Surprisingly, there is a notable absence of studies investigating the mental health status of construction workers in Bangladesh. Therefore, the primary objective of this study was to fill this gap by providing baseline data through an investigation into the prevalence and factors associated with depression, anxiety, and stress among construction workers in the country.

## Methods

### Study design, area, and participants

A cross-sectional study was conducted in a community setting from February 2022 to June 2022. This study surveyed 502 Bangladeshi construction workers across 40 construction sites in the metropolitan areas of Khulna, Jessore, Barisal, and Patuakhali. The districts were all located in the southern region of Bangladesh and the sites were selected based on the convenience of the data collection. Simple random sampling was used to select participants from the visited construction sites. A list of construction employees was made while visiting the locations. Random selection was used to choose the participants from the prepared list. There were no age restrictions for participants. We include construction workers with at least one year of experience. The study excluded construction employees who were unable to communicate adequately (due to hearing or communication impairments).

### Sample size estimation

The sample size for the study was calculated using the Cochran Formula (Cochran, 1977), $n=\frac{z^{2}pq}{d^{2}}$, where *n* is the required sample size, *z* is 1.96 at a 95% confidence interval, *d* is the margin of error at 5%, and *q* = *1–p*. The study was the first of its kind in Bangladesh, so a probability of 50% was used for expected prevalence, which was also used in other studies to estimate sample size [25, 26]. However, a total of 384 samples were required for this study, but 505 participants were surveyed, with three being excluded due to incomplete information, resulting in a final sample size of 502. This sample size was larger than the calculated sample size to ensure more precise and accurate results. All data were collected by expert interviewers in a bias-free environment, ensuring anonymity, providing equal access, and maintaining cultural sensitivity.

### Data collection and quality control

A standardized questionnaire was created and distributed to trained data collectors who were familiar with survey administration. The questionnaire was initially prepared in English, then translated into Bengali, and then back to English to ensure uniformity. Nine data collectors were trained for three days before conducting the surveys. To reduce variations in data collection, the definition of variables was standardized and stated to the data collectors. A pretest was done with a sample of 25 construction workers before the final study, and any uncertain questions were modified based on the results. Supervisors conducted daily evaluations of the responses to ensure uniformity and completeness.

Data was collected through interview-administered questionnaires. Interviewers discussed the study’s aims, respondent expectations, and the study’s risks and benefits before conducting the interview. Interviews were conducted before work, during a break, or after work to ensure clear understanding and responses. Supervisors actively supervised the data collection team to ensure accuracy and consistency.

### Predictor variables

The survey questionnaire included a range of variables such as socio-demographic information, lifestyle factors, occupational factors, and health hazards. The main outcome variables of this study were depression, anxiety, and stress. Other variables included age, marital status, monthly income, smoking status, sleep duration, types of construction work, working duration per day, duration of involvement in construction site, and working in dusty environments (including sand, cement, brick, rock, concrete, plasterboard, mortar, wood byproducts, and stone) [27], use of masks during work, presence of family members with breathing problems, and presence of breathing problems after starting construction work [28]. Sleep duration was classified into two categories: (a) <7 hours, (b) > = 7 hours [29]. Monthly income was divided into three categories: lower-class (<15,000 BDT), middle-class, and upper-class (>30,000 BDT), based on the previous study [30].

### Mental health problems

The Depression, Anxiety, and Stress Scale 21 [31] is an extensively utilized self-report instrument for screening depression, anxiety, and stress. For this study, the Bangla version of the scale was used [32]. This scale consists of 21 items measured on a four-point Likert scale comprising from never (0) to always (3) and provides scores for three subscales containing seven items each (depression, anxiety, and stress). In the present study, those with moderate to severe depression (≥14 on the depression subscale), anxiety (≥15 on the anxiety subscale), and stress (≥19 on the stress subscale) were evaluated. The Cronbach’s Alpha reliability coefficients for the DASS-21 were 0.84 for the overall scale, 0.74 for depression, 0.77 for anxiety, and 0.71 for stress.

### Statistical analysis

All statistical analyses were conducted using SPSS version 28.0. Descriptive statistics (frequency, mean, median, standard error, deviation, percentages, etc.) were used to summarize socio-demographic characteristics, lifestyle, occupational, and psychological factors. Chi-square tests, and unadjusted and adjusted logistics regression models were developed to assess the association of main outcomes (depression, anxiety, and stress) with independent variables. The multicollinearity of variables was also assessed before entering into regression analysis. The presence of multicollinearity among independent variables was checked using a correlation coefficient at the cutoff value of 0.8 [33]. All variables were entered into the multivariable analysis. Model fitness was checked using the Hosmer-Lemeshow goodness of fit test (*p* > 0.05). Odds ratio (ORs) and 95% confidence intervals were calculated for each variable included in the regression models with a significance *p*-value less than 0.05. All tests were done using SPSS version 28.0.

### Ethical approval and consent to participate

The study adhered to the ethical guidelines outlined in the Helsinki Declaration 2013, and was approved by the ethical review committee of the Dept. of Biochemistry and Food Analysis, Patuakhali Science and Technology University, Bangladesh (Approval Number: BFA: 17/01/2022:02). Every survey respondent was made aware of the study’s goals, the confidentiality of their information, how it will be used afterward, and their right to withdraw at any time. Participants were required to provide written informed consent to participate in the study. For the participants aged below 16 years, informed consent was obtained from their LAR or guardian.

## Results

**Fig 1:**
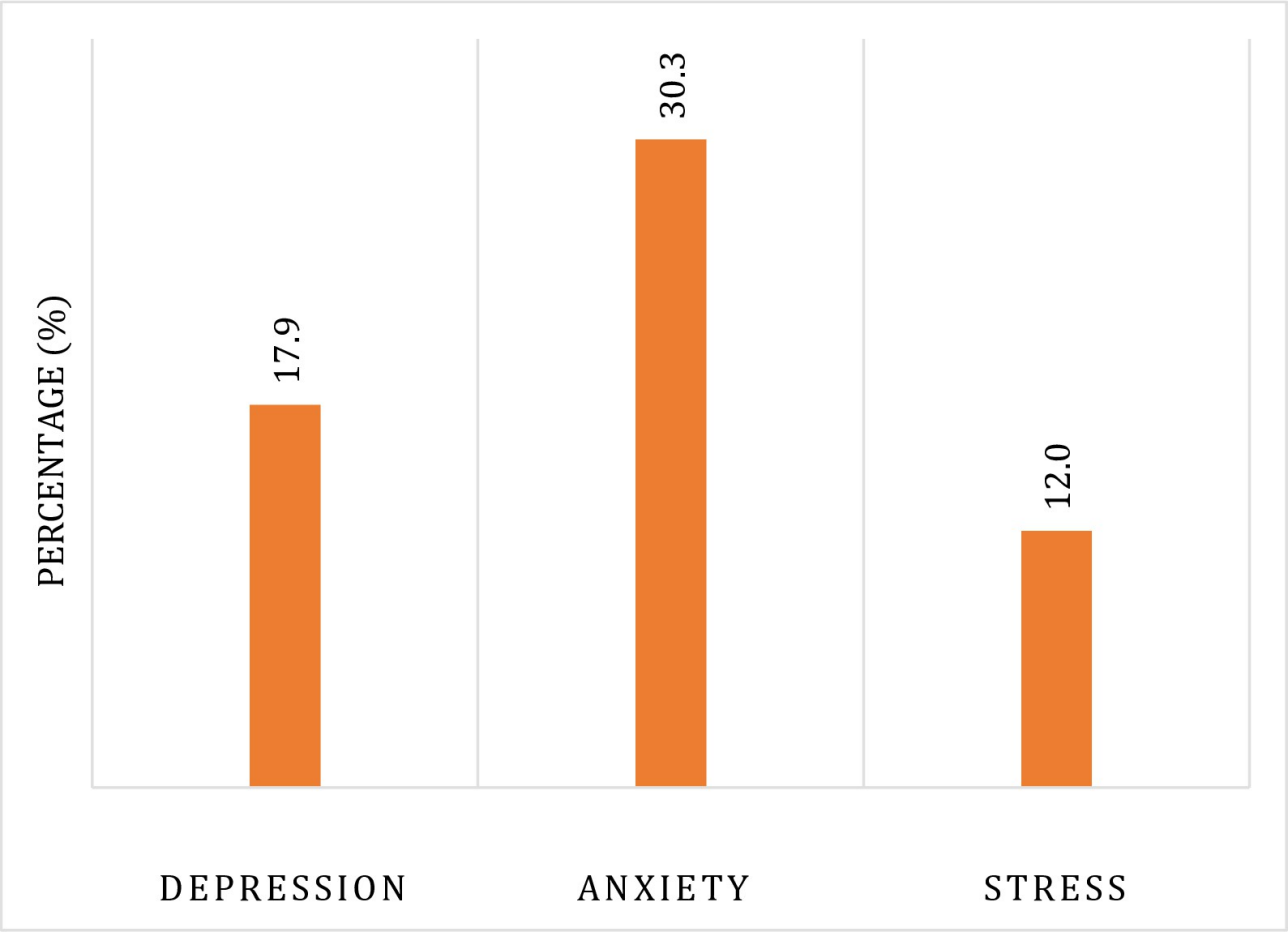
Prevalence of depression, anxiety, and stress among construction workers.

This study consisted of 502 construction employees with a mean age of [32.87±11.16]. Of most participants, 79.5% were married, 68.1% had monthly incomes less than 15000 BDT, and 59.8% smoked. Of the participants, 53.8% had healthy sleep duration, 28.5% had breathing problems after starting working in construction works and 7.2% had someone suffering from breathing problems in their family. About 47% of the participants reported working in manual labor, whereas 92.6% worked in dusty environments, and only 16.7% used masks at work (**Table 1**). The prevalence of depression, anxiety, and stress among construction workers was 17.9%, 30.3%, and 12%, respectively (**Fig 1**).

**Table 1:** Sociodemographic characteristics.

Variables & categories	n	%
**Age**		
18 or below	35	7.0
19–25	122	24.3
26–35	177	35.3
36–45	96	19.1
46 or up	72	14.3
**Marital status**		
Married	399	79.5
Single	103	20.5
**Monthly income (BDT)**		
<15000	342	68.1
15000 to30000	153	30.5
>30000	7	1.4
**Smoking status**		
Yes	300	59.8
No	202	40.2
**Sleep duration**		
≥7 hours	270	53.8
<7 hours	232	46.2
**Types of construction work**		
Manual labor	236	47.0
Bricklayer	79	15.7
Armature fixing	78	15.5
Internal finish worker	67	13.3
Painter and electrician	42	8.4
**Working duration per day**		
<8 hours	270	53.8
9–10 hours	219	43.6
>11 hours	13	2.6
**Involvement in construction site**		
<5 year	170	33.9
5–10 years	116	23.1
>10 years	216	43.0
**Working in the dusty environment**		
Yes	465	92.6
No	37	7.4
**Use a mask during working**		
Yes	84	16.7
No	418	83.3
**Have family member with breathing problem**		
Yes	36	7.2
No	466	92.8
**Have breathing problems after started working construction work**		
No	359	71.5
Yes	143	28.5

According to **Table 2**, certain factors were found to be significantly associated with depression. This included age (χ^2^ = 13.38, *p* = 0.010), sleep duration (χ^2^ = 14.66, *p*< 0.001), having breathing problems in the family (χ^2^ = 4.20, *p* = 0.040), and experiencing breathing problems after starting construction work (χ^2^ = 8.58, *p* = 0.003). Similarly, factors like age (χ^2^ = 9.89, *p* = 0.042), having breathing issues in the family (χ^2^ = 7.15, *p* = 0.008), and experiencing breathing issues after beginning construction work (χ^2^ = 69.38, *p*<0.001) appeared being associated with anxiety. Finally, breathing issues in the family (χ^2^ = 3.89, *p* = 0.049) and their breathing problems (χ^2^ = 13.18, *p*<0.001) were substantially linked to stress.

**Table 2:** Distribution of variables among construction workers by depression, anxiety, and stress.

Variables & categories	Depression (n = 90, 17.9%)				Anxiety (n = 152, 30.3%)				Stress (n = 60, 12%)			
	No (%)	Yes (%)	χ^2^	*p*-value	No (%)	Yes (%)	χ^2^	*p*-value	No (%)	Yes (%)	χ^2^	*p*-value
**Age**												
18 or below	31 (88.6)	4 (11.4)	13.38	**0.010**	27 (77.1)	8 (22.9)	9.89	**0.042**	34 (97.1)	1 (2.9)	7.41	0.116
19–25	91 (74.6)	31 (25.4)			91 (74.6	31 (25.4)			105 (86.1)	17 (13.9)		
26–35	157 (88.7)	20 (11.3)			129 (72.9)	48 (27.1)			161 (91.0)	16 (9.0)		
36–45	73 (76.0)	23 (24.0)			62 (64.6)	34 (35.4)			83 (86.5)	13 (13.5)		
46 or up	60 (83.3)	12 (16.7)			41 (56.9)	31 (43.1)			59 (81.9)	13 (18.1)		
**Marital status**												
Married	332 (83.2)	67 (16.8)	1.71	0.191	271 (67.9)	128 (32.1)	2.99	0.084	353 (88.5)	46 (11.5)	0.33	0.565
Single	80 (77.7)	23 (22.3)			79 (76.7)	24 (23.3)			89 (86.4)	14 (13.6)		
**Monthly income (BDT)**												
<15000	282 (82.5)	60 (17.5)	0.21	0.902	239 (69.9)	103 (30.1)	0.03	0.986	305 (89.2)	37 (10.8)	1.31	0.519
15000 to30000	124 (81.0)	29 (19.0)			106 (69.3)	47 (30.7)			131 (85.6)	22 (14.4)		
>30000	6 (85.7)	1 (14.3)			5 (71.4)	2 (28.6)			6 (85.7)	1 (14.3)		
**Smoking status**												
Yes	250 (83.3)	50 (16.7)	0.81	0.369	215 (71.7)	85 (28.3)	1.34	0.248	268 (89.3)	32 (10.7)	1.17	0.279
No	162 (80.2)	40 (19.8)			135 (66.8)	67 (33.2)			174 (86.1)	28 (13.9)		
**Sleep duration**												
≥7 hours	238 (88.1)	32 (11.9)	14.66	**<0.001**	191 (70.7)	79 (29.3)	0.29	0.592	241 (89.3)	29 (10.7)	0.82	0.367
<7 hours	174 (75.0)	58 (25.0)			159 (68.5)	73 (31. 5)			201 (86.6)	31 (13.4)		
**Types of construction work**												
Manual labor	183 (77.5)	53 (22.5)	7.07	0.132	163 (69.1)	73 (30.9)	0.59	0.964	209 (88.6)	27 (11.4)	3.69	0.450
Bricklayer	70 (88.6)	9 (11.4)			55 (69.6)	24 (30.4)			73 (92.4)	6 (7.6)		
Armature fixing	66 (84.6)	12 (15.4)			53 (67.9)	25 (32.1)			68 (87.2)	10 (12.8)		
Internal finish worker	56 (83.6)	11 (16.4)			49 (73.1)	18 (26.9)			58 (86.6)	9 (13.4)		
Painter and electrician	37 (88.1)	5 (11.9)			30 (71.4)	12 (28.6)			34 (81.0)	8 (19.0)		
**Working duration per day**												
<8 hours	230 (85.2)	40 (14.8)	5.65	0.059	189 (70.0)	81 (30.0)	0.39	0.822	245 (90.7)	25 (9.3)	4.03	0.134
9–10 hours	170 (77.6)	49 (22.4)			151 (68.9)	68 (31.1)			186 (84.9)	33 (15.1)		
>11 hours	12 (92.3)	1 (7.7)			10 (76.9)	3 (23.1)			11 (84.6)	2 (15.4)		
**Involvement in construction site**												
<5 year	132 (77.6)	38 (22.4)	3.72	0.156	125 (73.5)	45 (26.5)	3.59	0.166	151 (88.8)	19 (11.2)	0.20	0.903
5–10 years	96 (82.8)	20 (17.2)			84 (72.4)	32 (27.6)			101 (87.1)	15 (12.9)		
>10 years	184(85.2)	32 (14.8)			141 (65.3)	75 (34.7)			190 (88.0)	26 (12.0)		
**Working in the dusty environment**												
Yes	385 (82.8)	80 (17.2)	2.25	0.134	322 (69.2)	143 (30.8)	0.67	0.413	407 (87.5)	58 (12.5)	1.63	0.202
No	27 (73.0)	10 (27.0)			28 (75.7)	9 (24.3)			35 (94.6)	2 (5.4)		
**Use a mask during working**												
Yes	70 (83.3)	14 (16.7)	0.11	0.741	66 (78.6)	18 (21.4)	3.74	0.053	70 (83.3)	14 (16.7)	2.13	0.144
No	342 (81.8)	76 (18.2)			284 (67.9)	134 (32.1)			372 (89.0)	46 (11.0)		
**Have family member with breathing problem**												
Yes	25 (69.4)	11 (30.6)	4.20	**0.040**	18 (50.0)	18 (50.0)	7.15	**0.008**	28 (77.8)	8 (22.2)	3.89	**0.049**
No	387 (83.0)	79 (17.0)			332 (71.2)	134 (28.8)			414 (88.8)	52 (11.2)		
**Have breathing problems after started working construction work**												
No	306 (85.2)	53 (14.8)	8.58	**0.003**	289 (80.5)	70 (19.5)	69.38	**<0.001**	328 (91.4)	31 (8.6)	13.18	**<0.001**
Yes	106 (74.1)	37 (25.9)			61 (42.7)	82 (57.3)			114 (79.7)	29 (20.3)		

The adjusted logistic regression model in Table 3 demonstrates a well-fitting model, with p-values of 0.326, 0.528, and 0.629 for depression, anxiety, and stress, respectively., Respondents who had healthy sleep duration were 64% (AOR = 0.36; 95% CI: 0.21–0.61) less likely to be depressed compared to satisfied respondents. Employees who did not experience respiratory issues after beginning their construction work were 46% (AOR = 0.55; 95% CI: 0.32–0.97) less likely to experience depression than those who did. When compared to workers who had breathing issues after starting construction work, those who did not have these issues were 45% (AOR = 0.18; 95% CI: 0.11–0.30) less likely to experience anxiety. Respondents under the age of 18 were 94% (AOR = 0.06; 95%Cl: 0.01–0.69) less likely than respondents in the older age group to report experiencing stress. Compared to painters and electricians, bricklayer employees were 72% (AOR = 0.28; 95% CI: 0.08–0.95) less likely to experience stress. Stress increased 3.03 times when wearing a mask at work (AOR = 3.03; 95%Cl: 1.40–6.53). When compared to workers who had breathing issues after beginning their construction work, those without breathing issues were 66% (AOR = 0.34; 95% CI: 0.17–0.66) less likely to experience stress.

**Table 3:** Binary regression analysis of the variables by depression, anxiety, and stress.

Variables & categories	Depression		Anxiety		Stress	
	AOR [95% CI]	*p*-value	AOR [95% CI]	*p*-value	AOR [95% CI]	*p*-value
**Age**						
18 or below	0.26 [0.05–1.25]	0.092	0.68 [0.18–2.66]	0.582	0.06 [0.01–0.69]	**0.024**
19–25	0.84 [0.29–2.46]	0.754	0.66 [0.26–1.68]	0.387	0.48 [0.14–1.64]	0.243
26–35	0.52 [0.21–1.31]	0.166	0.52 [0.26–1.06]	0.070	0.43 [0.16–1.11]	0.081
36–45	1.35 [0.57–3.20]	0.503	0.65 [0.32–1.32]	0.228	0.66 [0.26–1.67]	0.375
46 or up	Reference		Reference		Reference	
**Marital status**						
Married	0.72 [0.34–1.55]	0.400	1.38 [0.63–3.02]	0.416	0.39 [0.15–1.02]	0.055
Single	Reference		Reference		Reference	
**Monthly income (BDT)**						
<15000	1.50 [0.13–17.52]	0.748	2.79 [0.42–18.71]	0.291	1.77 [0.15–20.31]	0.646
15000 to30000	1.81 [0.15–21.47]	0.640	2.63 [0.39–17.70]	0.321	2.20 [0.19–25.86]	0.530
>30000	Reference		Reference		Reference	
**Smoking status**						
Yes	0.76 [0.44–1.30]	0.319	0.89 [0.56–1.41]	0.625	0.83 [0.44–1.55]	0.561
No	Reference		Reference		Reference	
**Sleep duration**						
≥7 hours	0.36 [0.21–0.61]	**<0.001**	1.01 [0.65–1.56]	0.980	0.82 [0.44–1.51]	0.518
<7 hours	Reference		Reference		Reference	
**Types of construction work**						
Manual labor	2.05 [0.71–5.93]	0.185	0.90 [0.40–2.03]	0.803	0.45 [0.17–1.19]	0.107
Bricklayer	0.81 [0.23–2.78]	0.731	1.02 [0.41–2.53]	0.970	0.28 [0.08–0.95]	**0.041**
Armature fixing	1.13 [0.34–3.78]	0.846	0.96 [0.39–2.38]	0.932	0.44 [0.14–1.35]	0.151
Internal finish worker	1.19 [0.35–4.07]	0.779	0.70 [0.27–1.83]	0.466	0.48 [0.15–1.52]	0.210
Painter and electrician	Reference		Reference		Reference	
**Working duration per day**						
<8 hours	2.20 [0.26–18.75]	0.472	1.76 [0.40–7.73]	0.457	0.82 [0.15–4.44]	0.813
9–10 hours	3.08 [0.37–26.04]	0.301	1.45 [0.33–6.32]	0.625	1.30 [0.24–6.91]	0.760
>11 hours	Reference		Reference		Reference	
**Involvement in construction site**						
<5 year	2.18 [0.98–4.84]	0.057	0.93 [0.47–1.85]	0.833	1.23 [0.48–3.12]	0.669
5–10 years	1.61 [0.76–3.42]	0.215	1.04 [0.56–1.92]	0.898	1.42 [0.60–3.33]	0.425
>10 years	Reference		Reference		Reference	
**Working in the dusty environment**						
Yes	0.63 [0.27–1.49]	0.295	0.77 [0.33–1.81]	0.551	3.47 [0.71–16.98]	0.125
No	Reference		Reference		Reference	
**Use a mask during working**						
Yes	1.23 [0.61–2.51]	0.562	1.04 [0.57–1.93]	0.893	3.03 [1.40–6.53]	**0.005**
No	Reference		Reference		Reference	
**Have family member with breathing problem**						
Yes	1.89 [0.80–4.42]	0.145	1.28 [0.59–2.80]	0.531	1.63 [0.65–4.12]	0.299
No	Reference	.	Reference	.	Reference	
**Have breathing problems after started working construction work**						
No	0.55 [0.32–0.97]	**0.037**	0.18 [0.11–0.30]	**<0.001**	0.34 [0.17–0.66]	**0.001**
Yes	Reference		Reference	.	Reference	

AOR = Adjusted odds ratio, CI = Confidence interval

## Discussion

The primary objective of this study was to assess the prevalence and associated factors of depression, anxiety, and stress among construction workers in Bangladesh. Our investigation unveiled that the prevalence rates of depression, anxiety, and stress stood at 17.9%, 30.3%, and 12% respectively, figures that were notably lower than those reported in other Bangladeshi cohorts [34–36]. However, a significant finding emerged regarding the association between breathing issues subsequent to engaging in construction work and mental health problems. Specifically, experiencing respiratory problems following involvement in construction activities emerged as a significant risk factor for mental health challenges. This highlights the critical importance of addressing respiratory health in the context of occupational well-being among construction workers.

Construction workers are known to have higher rates of mental health problems than other occupations. This has been demonstrated in multiple studies from different countries, including the United States, Netherlands, Korea, and Vietnam. For example, in the United States, manual laborers who could not work due to injuries were likelier to report higher levels of depression (17.7%) [37]. This is similar to the prevalence found in the Netherlands (17.6%) [8]. In Korea and Vietnam, even higher rates have been documented among construction workers (37.6% and 25%, respectively) [13, 14]. However, it’s worth noting that there are some differences in the rate of anxiety and stress among construction workers. For example, the prevalence of anxiety among Ghanaian construction workers was 14.2%, which is lower than this study [38]. On the other hand, the prevalence of anxiety among Indian construction workers was 93.4%, which is higher than this study [39]. It’s possible that these differences can be attributed to a variety of factors, such as participant age, sampling, research schedule, cultural influences, and study context. Additionally, the instruments used to measure depression, anxiety, and stress may have also contributed to the observed disparities. Unfortunately, there is no other relevant research among construction workers in Bangladesh, so it is impossible to make more thorough comparisons to clarify temporal trends or additional relevant factors.

The intricate interplay between sleep problems and mental health issues among construction workers highlighting the pressing need for targeted interventions in this occupational sector. Numerous studies have consistently reported the heightened risk of sleep disturbances among individuals working in construction, with these disruptions significantly associated with increased vulnerability to depression and anxiety. Indeed, depression is alarmingly prevalent within the construction workforce, with poor sleep quality serving as a prominent contributing factor. This assertion finds support in comprehensive systematic review and meta-analysis [40], corroborating the pervasive link between sleep disturbances and mental health challenges in this demographic. Furthermore, our study’s findings align with previous research, reaffirming the detrimental impact of poor sleep quality on the development of depressive symptoms [41]. For instance, longitudinal investigations conducted among Japanese male employees found that those experiencing sleep deprivation were nearly seven times more likely to exhibit symptoms of depression [41]. Moreover, individuals grappling with sleeplessness reported heightened levels of stress and depression, irrespective of their working schedules [42]. These findings emphasis the critical imperative for occupational health care professionals to address the issue of poor sleep quality among construction workers promptly. To mitigate the adverse effects of poor sleep on mental well-being within the construction industry, multifaceted treatment approaches must be implemented. These interventions should be tailored to address the unique challenges faced by construction workers, focusing on fostering improved sleep hygiene practices and reducing the incidence of depression. Implementing comprehensive interventions that encompass various components, such as cognitive-behavioral therapy, mindfulness techniques, and workplace accommodations, holds promise in promoting better sleep and safeguarding the mental health of construction workers. By prioritizing interventions that target sleep quality, stakeholders can proactively mitigate the risk of depression and enhance the overall well-being of this vital workforce.

There is a complex relationship between breathing problems and mental health. Studies have found that individuals with respiratory symptoms such as asthma and shortness of breath are at an increased risk for developing mental health problems, particularly anxiety and depression. A systematic review and meta-analysis found that individuals with asthma were more likely to have symptoms of depression compared to those without asthma [43], and that is also consistent with other breathing-related issues such as asthma and COPD. Some studies suggest a connection between psychological discomfort and respiratory issues [44], with anxiety and depression linked to respiratory symptoms but not necessarily asthma or bronchial reactivity [45]. Research also shows that poorly managed asthma and respiratory symptoms such as shortness of breath can lead to an anxiety disorder [46]. As per this study’s findings, breathing problems after being engaged in construction work were found to be the predictive factors of mental health problems. However, the underlying mechanisms of this relationship are not fully understood. Studies examining this relationship have yielded mixed results, with some showing a strong link and others only a weak one [47, 48]. Further investigation is needed to better understand and address the connection between respiratory issues and mental health, especially for construction workers.

Older construction workers may be at a greater risk for poor mental health due to their increased vulnerability to physical and environmental hazards. According to the findings of this study, participants who were 46 years old or older had a much higher risk of poor mental health (although depression and anxiety are not significant) than those who were younger. Despite the demanding nature of construction work, older workers may be more susceptible to poor mental health due to exposure to high temperatures, awkward postures, manual material handling, and other hazards [49]. Similarly, a previous study indicates that older construction workers are at a greater risk for poor mental health, suggesting that complete assistance is required to address working conditions and psychological health issues [49]. Besides, younger construction workers may also be at risk for poor mental health due to high job expectations and lack of workplace support. These individuals may feel the need to "prove themselves" and may be uncertain about their organizational position and available assistance [50]. Thus, it is important for the construction industry to address not only working conditions but also psychological health issues for of all ages construction workers.

This study has limitations, such as the inclusion of only male employees and data collected from a few specific locations that were conveniently selected; there is a possibility of biased results. Hence, a nationwide sample, including both genders, would be considered in further studies. The findings cannot establish causality but rather show a cross-sectional relationship. In addition, there may have been a lack of incentives for participation and enthusiasm during the question-answer period. However, it also has strengths, such as being one of the most recent studies to determine the prevalence and factors influencing depression, anxiety, and stress among Bangladeshi construction workers. The findings can aid decision-makers in improving the health and safety of construction workers.

## Conclusions

This study is the first of its kind to examine the mental health of construction workers in Bangladesh. These findings can be utilized to improve public health policies and occupational health standards in the country, reducing the prevalence of mental health problems and protecting the country’s labor force. Key risk factors identified include poor sleep quality and the onset of breathing problems after beginning construction work, both of which significantly contribute to the likelihood of experiencing mental health issues. Working as bricklayers and those experiencing breathing issues are particularly vulnerable to stress, highlighting the necessity for targeted interventions. However, it is important to note that this study highlights the need for more research on the mental health of construction workers and their impact on their work and well-being. This population is likely underreported and hesitant to seek professional help for their mental health problems. It also suggests that this high-risk group requires more treatment options, education, and acceptance of mental disorders. Further research is necessary to determine feasible intervention methods that can be implemented to improve the mental health of construction workers in Bangladesh. With the help of these findings, better measures can be taken to safeguard the mental well-being of this essential workforce, which will positively impact their productivity and the overall growth of the country.

## Supporting information

S1 Dataset Dataset used for analyses in present study. (XLSX)
